# Evaluation of magnetic resonance imaging for bladder cancer detection following transurethral resection of bladder tumour (TURBT)

**DOI:** 10.1007/s00261-024-04235-6

**Published:** 2024-05-08

**Authors:** Samir A. Khwaja, Iztok Caglic, Nadeem Shaida, Alexandra J. Colquhoun, William Turner, Tristan Barrett

**Affiliations:** 1 Department of Radiology, Addenbrooke’s Hospital and University of Cambridge, Hills Road, Cambridge, CB2 0QQ UK; 2 Department of Urology, Addenbrooke’s Hospital, Hills Road, Cambridge, CB2 2QQ UK

**Keywords:** Bladder cancer, Magnetic resonance imaging, Transurethral resection of bladder tumour (TURBT), Diffusion weighted imaging (DWI), Staging

## Abstract

**Purpose:**

To evaluate the performance of MRI for detection of bladder cancer following transurethral resection of bladder tumour (TURBT).

**Methods:**

This single-centre retrospective study included forty-one consecutive patients with bladder cancer who underwent bladder MRI after TURBT. Two uroradiologists retrospectively assessed the presence of tumour using bladder MRI with and without DWI (diffusion weighted imaging) using a five-point Likert scale. Sensitivity, specificity, positive predictive value (PPV) and negative predictive value (NPV) were calculated and inter-reader agreement was assessed. Histopathology was used as the reference standard.

**Results:**

24 out of 41 patients (58.5%) had no residual tumour or Tis (carcinoma in situ) after TURBT. Sensitivity, specificity, PPV and NPV for detection of tumour using T1WI (T1-weighted imaging) and T2WI (T2-weighted imaging) was 50.0%, 54.6%, 21.1%, and 81.8%, respectively and for T1WI, T2WI and DWI combined was 100%, 76.5%, 50.0% and 100%, respectively. Overestimation of tumour was more common than underestimation. MRI showed high accuracy for patients in whom there was no residual tumour (78.9%). Inter-reader agreement for tumour detection improved from fair (κ = 0.54) to moderate (κ = 0.70) when DWI was included.

**Conclusion:**

Non-contrast MRI with DWI showed high sensitivity and relatively high specificity for detection of residual tumour after TURBT. Inter-reader agreement improved from fair to moderate with the addition of DWI. MRI can be useful after TURBT in order to guide further management.

**Graphical abstract:**

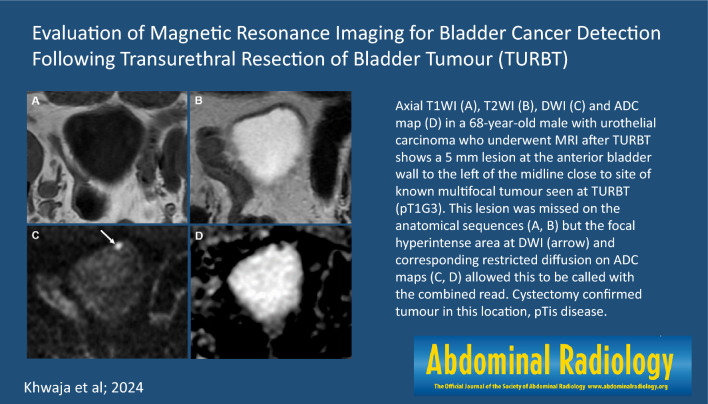

**Supplementary Information:**

The online version contains supplementary material available at 10.1007/s00261-024-04235-6.

## Introduction

Globally bladder cancer is the tenth most common cause of cancer related death in men [1]. The majority of bladder cancers are urothelial carcinomas. Accurate pre-operative staging is essential to guide management of bladder cancer [2]. Approximately 75% of bladder tumours are non-muscle invasive cancers (NMIBC) which are commonly diagnosed and treated with local therapy by transurethral resection of bladder tumour (TURBT) and/or intravesical therapies [3, 4]. Muscle-invasive bladder cancer (MIBC) is typically treated by radical cystectomy or radiation therapy [5].

In the UK national timed-cancer pathway targets mandate that for at least 85% of patients, cancer treatment commences within 62 days of referral [6]. The updated 2015 national cancer strategy proposed a 28-day target in which to confirm or rule out a cancer diagnosis following urgent referral with a suspicion of cancer [7]. Current diagnostic pathways for bladder cancer rely on cystoscopy and CT or MRI followed by TURBT [8]. TURBT is regarded both as a form of definitive treatment and local staging for NMIBC, however studies have shown that TURBT can underestimate T-categorisation in up to 40% of patients [9, 10]. Patients with MIBC who are under-staged are at significantly higher risk for disease progression and worse outcomes, with an approximately 30% higher five-year cancer specific mortality [10].

Data from Getting It Right First Time (GIRFT), an NHS (National Health Service) programme designed to improve quality of care in urology, showed that in 2013–2016 patients with MIBC were waiting on average of 144 days from referral to cystectomy [11]. One of the contributory factors to this delay is the wait for imaging investigations. Although bladder MRI is the cornerstone for local staging and is recommended by international guidelines, our experience is that MRI is generally underutilised in the UK due to pressures to meet timed-cancer pathway targets. Scheduling and performing bladder MRI between cystoscopy and TURBT can be challenging with MRI often performed after intervention. Preliminary data from an ongoing randomised trial (BladderPath) comparing risk-stratified (5-point Likert scale) image-directed care with TURBT for newly diagnosed bladder cancer suggest that it is feasible to direct MIBC patients to multiparametric MRI for staging instead of TURBT, thereby reducing time to treatment [12].

The VI-RADS (Vesical Imaging Reporting and Data System) scoring system has helped standardise MRI acquisition and reporting of bladder cancer, with several studies demonstrating good diagnostic performance and reproducibility for assessment of MIBC [13, 14]. However, interpretation of MRI after TURBT can be challenging due to the presence of inflammation and fibrosis within the bladder wall or extravesical fat. VI-RADS is not intended to be used post-TURBT and few studies have investigated the diagnostic performance of MRI in this setting. El-Assmy et al. demonstrated that DWI (diffusion weighted imaging) can reliably differentiate post-TURBT inflammatory change from tumour with results similar to those of conventional cystoscopy [15]. van der Pol et al. found that multiparametric MRI showed high sensitivity and specificity for detection of MIBC, however the inclusion of only patients who underwent cystectomy is likely to have led to a selection bias [16]. Lim et al. showed that MRI with textural analysis can help in the local staging of bladder cancer after TURBT, however this technique is yet to enter routine clinical practice [17]. No further studies have been performed to date. We therefore aimed to assess the diagnostic performance of MRI for detection of bladder cancer following TURBT using histopathology as a reference standard.

## Methods

**Fig.1:**
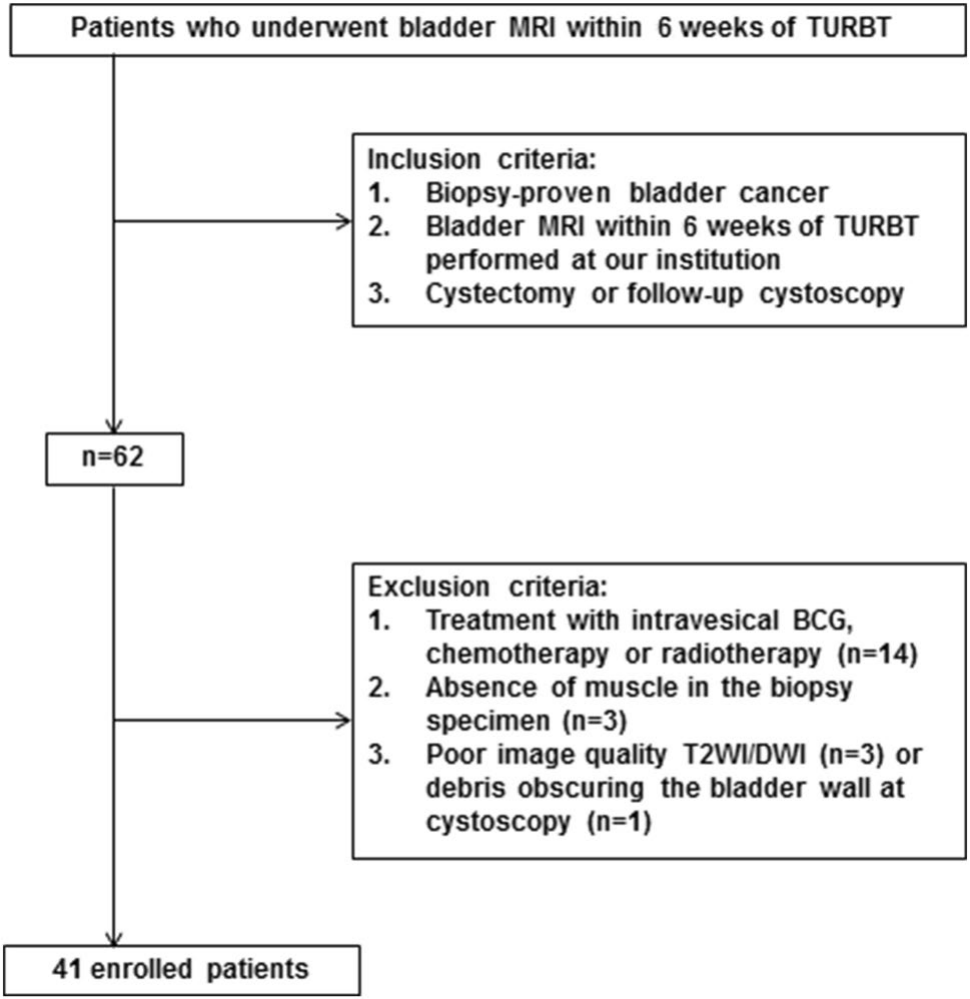
Flow diagram showing patient enrolment

Between August 2016 and January 2018, consecutive patients who underwent MRI post cystoscopy for bladder cancer at our institution and meeting study eligibility criteria were identified. The local institutional ethics committee approved this retrospective study, with the need to obtain informed consent waived (NRES Committee East of England, UK, reference A093248). The inclusion criteria were (a) biopsy-proven bladder cancer, (b) bladder MRI including T1-weighted images (T1WI), T2-weighted images (T2WI) and DWI within 6 weeks of TURBT performed at our institution and (c) either cystectomy or follow-up cystoscopy within 90 days. 62 patients met the initial inclusion criteria, with 21 patients excluded due to concurrent treatment with intravesical BCG, systemic chemotherapy or radiotherapy (n = 14), absence of muscle in the biopsy specimen (n = 3), poor quality T2WI/DWI owing to motion artefact or hip prosthesis (n = 3), or debris obscuring the bladder wall at cystoscopy (n = 1), giving a final eligible study cohort of 41 patients (Fig. 1).

### MRI protocol

Patients underwent bladder MRI using a 1.5 T MR450 (GE Healthcare, Waukesha, WI, USA) with a 16-channel phased array body coil. Unless contraindicated, intravenous injection of hyoscine butylbromide (Buscopan, 20 mg/ml, Boehringer, Germany) was administered prior to imaging to reduce peristaltic movement. Conventional T1-weighted spin-echo images (449 ms repetition time (TR), 9 ms echo time (TE); 352 × 224 matrix; 24 × 24 cm field-of-view (FOV); 5 mm slice thickness; 2.5 mm intersection gap; one signal acquired) in the axial plane. High-resolution T2 weighted turbo spin-echo images (3700 ms TR, 86 ms TE; 320 × 288 matrix; 24 × 24 cm field-of-view (FOV); 3 mm slice thickness; 1 mm intersection gap; two signals were acquired. Images were obtained in three orthogonal planes. Diffusion-weighted imaging (DWI) was performed using a spin-echo echo-planar imaging pulse sequence (5000 ms TR, 78 ms TE; 128 × 128 matrix; 27 × 27 cm field-of-view (FOV); 4.5 mm slice thickness; 0 mm intersection gap with b-values of 0 and 1000 s/mm^2^. Apparent diffusion coefficient (ADC) maps were calculated automatically.

### Image analysis

MR images were retrospectively evaluated by two subspecialist uroradiologists with 12 years (TB) and 6 years (NS) of experience in reading bladder MRI. Both readers reviewed MRI studies independently and were blinded to clinical data and pathology reports. Each reader independently assessed T1WI and T2WI together and T1WI, T2WI and DWI combined and assigned the perceived likelihood of tumour using a Likert scale: 1 = definitely absent, 2 = possibly absent, 3 = indeterminate, 4 = possibly present and 5 = definitely present. For each lesion size, location and T-categorisation was evaluated using the 8th edition of the American Joint Committee on Cancer (AJCC) staging manual [18]. In brief, readers assessed T1WI to look for haematoma, T2WI to look for a continuous low signal intensity line in the bladder wall that represents an intact muscularis propria and DWI (high *b*-value images) for tumour which is hyperintense with corresponding hypointensity on ADC maps. To minimise learning bias the second reading session was performed after a wash-out interval of 4 weeks. Readers only had access to specific sequences when reviewing each set of images. Differences in opinion were resolved by consensus, with the most experienced reader’s opinion considered as definitive.

### Histopathologic analysis

All biopsies and surgical specimens were analysed and graded according to existing guidelines [19] by an experienced uropathologist and reviewed by a second uropathologist at a multidisciplinary team meeting. Tumour cell type, and T-category were also obtained. In patients with multiple tumours the tumour with the highest pathological T-category was recorded.

### Data analysis

Demographic characteristics, MRI data and pathologic data were described with summary statistics. Sensitivity, specificity, positive predictive value (PPV), and negative predictive (NPV) value for detection of tumour using the Likert scale were calculated for both readers, using a 2 × 2 contingency table. For the purpose of data analysis, Likert score 3 was considered as positive for tumour as this reflects how this group of patients would be managed in clinical practice. Accuracy of MRI for T-categorisation was determined for for NMIBC detectable at MRI (< pT2, excluding pTis) and MIBC (≥ pT2). Data was analysed with the statistical package MedCalc (Version 20.305). The receiver operating characteristic (ROC) analysis was performed. Inter-reader agreement was analysed by means of weighted κ values with quadratic weighting (κ = 0.00–0.20, poor; 0.21–0.40, fair; 0.41–0.60, moderate; 0.61–0.80, good; and 0.81–1.00, excellent agreement).

## Results

41 patients were included in the study, 35 men (85.4%) and 6 women (14.6%) with median age of 74 years (IQR: 68–79 years). The median interval between TURBT and MRI was 26 days (IQR: 22–30 days). The median interval between MRI and follow-up cystoscopy or cystectomy was 16 days (IQR: 6–32 days).

### Histopathology

All patients had biopsy-proven bladder cancer at presentation. 40 patients had urothelial carcinoma and one patient had squamous cell carcinoma. At initial diagnosis all patients were classified as high-grade according to the WHO 2004 classification [20]. The histopathological reference was TURBT (n = 28) or cystectomy (n = 12). One patient underwent follow-up cystoscopy without biopsy followed by close clinical surveillance for ≥ 12 months to establish a benign diagnosis. In this study cohort, 58.5% (n = 24) had no residual tumour, 22.0% (n = 9) had pTis disease, 4.9% (n = 2) had pTa disease, 4.9% (n = 2) had pT1 disease, 4.9% (n = 2) had pT2 disease, 4.9% (n = 2) had pT3 disease and no patients had pT4 disease.

### Diagnostic performance of MRI for tumour detection

**Fig. 2:**
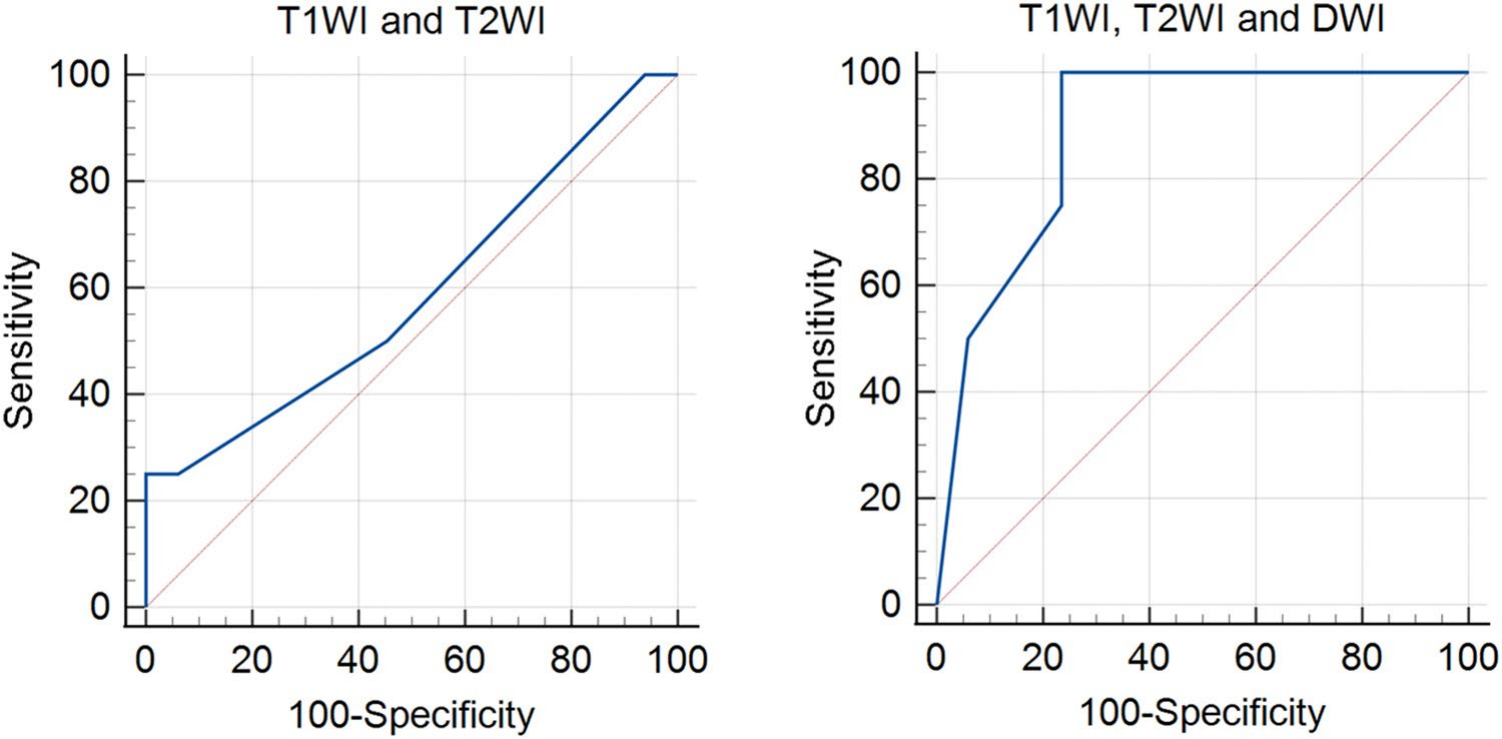
ROC curves for detection of bladder cancer following TURBT. AUC value of ROC analysis was 0.587 for T1WI and T2WI and 0.890 for T1WI, T2WI and DWI

Using T1WI and T2WI, consensus read identified 19 patients with one tumour and 22 patients with no tumour. Using T1WI, T2WI and DWI, consensus read identified one patient with two tumours, 15 patients with one tumour and 25 patients with no tumour. The sensitivity, specificity, PPV and NPV for detection of tumour using T1WI, T2WI were 50.0%, 54.6%, 21.1%, and 81.8%, respectively and compared to T1WI, T2WI and DWI at 100%, 76.5%, 50.0% and 100% respectively (Table 1). For both readers, using all three sequences (T1WI, T2WI and DWI) resulted in higher sensitivities, specificities, PPVs and NPVs compared to using T1WI and T2WI alone (Supplementary data Table 1). The area under the curve (AUC) value of ROC analysis was 0.587 for T1WI and T2WI and 0.890 for T1WI, T2WI and DWI (Fig. 2). Inter-reader agreement using T1WI and T2WI alone was 0.54 (95% CI 0.23–0.84) and improved to 0.70 (95% CI 0.48–0.91) using all three sequences.

**Table 1:** Diagnostic performance of MRI for tumour detection with T1WI and T2WI together and T1WI, T2WI and DWI combined

	T1WI and T2WI	T1W1, T2W1 and DWI
Sensitivity	50.0 (17.4−82.6)	100 (59.8−100)
Specificity	54.6 (36.6−71.5)	76.5 (58.4−88.6)
PPV	21.1 (7.0−46.1)	50.0 (25.5−74.5)
NPV	81.8 (59.0−94.0)	100 (84.0−100)

**Fig. 3:**
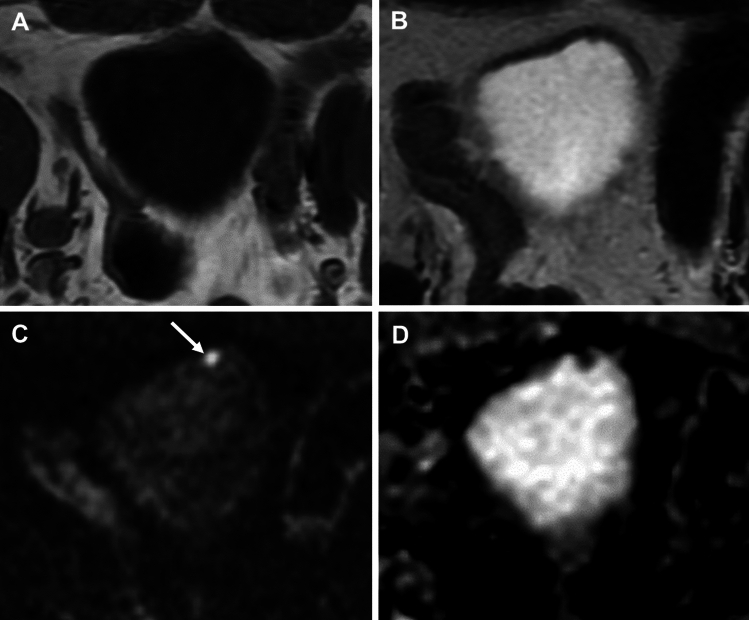
Axial T1WI (**A**), T2WI (**B**), DWI (**C**) and ADC map (**D**) in a 68-year-old male with urothelial carcinoma who underwent MRI after TURBT shows a 5 mm lesion at the anterior bladder wall to the left of the midline close to site of known multifocal tumour seen at TURBT (pT1G3). This lesion was missed on the anatomical sequences (**A**, **B**) but the focal hyperintense area at DWI (arrow) and corresponding restricted diffusion on ADC maps (**C**, **D**) allowed this to be called with the combined read. Cystectomy confirmed tumour in this location, pTis disease

**Fig. 4:**
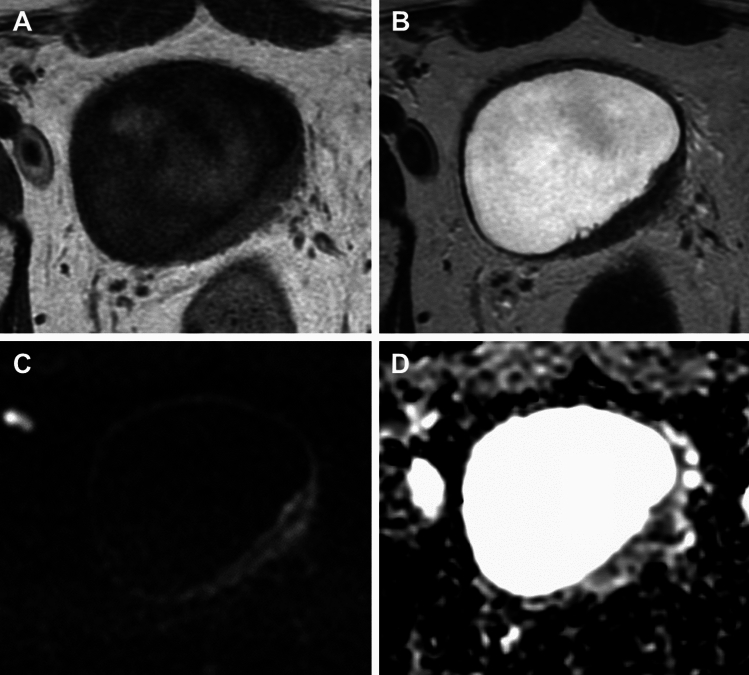
Axial T1WI (**A**), T2WI (**B**), DWI (**C**) and ADC map (**D**) in a 75-year-old male with urothelial carcinoma who underwent MRI after TURBT shows thickening and perivesical stranding at the left posterolateral bladder wall post-TURBT. Based on the anatomical sequences this finding could be interpreted as T3b disease (**A**, **B**), however there was no diffusion restriction to suggest residual/recurrence disease (**C**, **D**), the staging was therefore organ-confined disease. Cystectomy showed no residual disease.

**Fig. 5:**
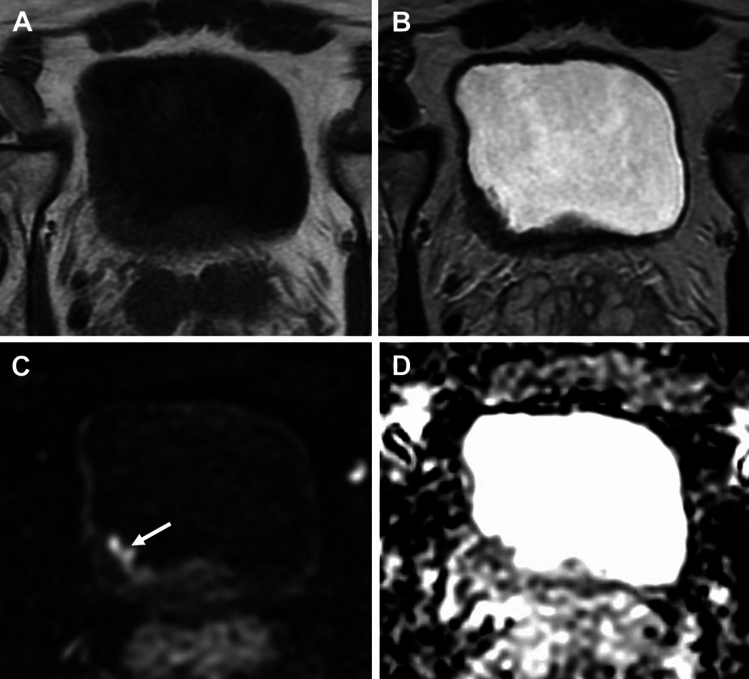
Axial T1WI (**A**), T2WI (**B**), DWI (**C**) and ADC map (**D**) in a 67-year-old male with urothelial carcinoma who underwent MRI after TURBT shows a 9 mm lesion at the right ureteric orifice post-TURBT (G3pTa). Based on the anatomical sequences this finding could be interpreted as residual disease (**A**, **B**). Although this was hyperintense at DWI (arrow) there was no diffusion restriction (**C**, **D**) as ADC was not low, and therefore this was interpreted as negative for residual/recurrence disease. Serial cystoscopic biopsies showed no evidence of malignancy

Overestimation of tumour was more common than underestimation (Table 2). The inclusion of DWI improved overall accuracy of MRI from 53.7% to 81.0%. Accuracy of MRI (with DWI) for detection of no tumour was 78.9% (26/34), 100% (4/4) for NMIBC and 100% (4/4) for MIBC. Case examples are shown in Figs. 3, 4 and 5.

**Table 2:** Diagnostic performance of MRI for tumour detection for each pathological T-category with T1WI and T2WI together and T1WI, T2WI and DWI combined

	T1WI and T2WI			T1WI,T2WI and DWI		
	Overestimation	Underestimation	Concordance	Overestimation	Underestimation	Concordance
pT0/Tis	36.6 (15)	−	43.9 (18)	19.0 (8)	−	61.9 (26)
pTa	0 (0)	2.4 (1)	2.4 (1)	0 (0)	0 (0)	4.8 (2)
pT1	0 (0)	2.4 (1)	2.4 (1)	0 (0)	0 (0)	4.8 (2)
pT2	0 (0)	2.4 (1)	2.4 (1)	0 (0)	0 (0)	4.8 (2)
pT3	0 (0)	2.4 (1)	2.4 (1)	0 (0)	0 (0)	4.8 (2)
pT4	−	0 (0)	0 (0)	−	0 (0)	0 (0)
	36.6 (15)	9.8 (4)	53.7 (22)	19.0 (8)	0 (0)	81.0 (34)

### Diagnostic performance of MRI for T-categorisation

For NMIBC detectable at MRI (< pT2, excluding pTis) accuracy of T-categorisation improved from 50% (2/4) to 100% (4/4) with DWI. For MIBC (≥ pT2) accuracy of T-categorisation improved from 50% (2/4) to 100% (4/4) with DWI.

## Discussion

In this study we assessed the diagnostic performance of MRI for tumour detection and T-categorisation. We found that MRI had high sensitivity, relatively high specificity and high NPV for tumour detection following TURBT. MRI showed high accuracy for patients with no residual tumour. However, overall PPV was only moderate, highlighting the limitations of MRI assessment in this setting and importance of close follow-up cystoscopic surveillance. Overestimation of tumour was more common than underestimation.

In our cohort MRI was performed at a median time of 26 days after TURBT. This was due to time constraints imposed by cancer treatment referral pathways and may compromise staging assessment. Despite the availability of non-invasive imaging and the risk of complications from multiple TURBTs, current guidelines do not specifically address whether and when to use MRI in this setting due to limited data.

To date, there have been limited studies investigating the diagnostic performance of bladder MRI post cystoscopic intervention. Van der Pol et al. reported high sensitivity and specificity for detection of MIBC in 45 patients who underwent TURBT followed by radical cystectomy [16]. More recently, Pecoraro et al. demonstrated the feasibility of a novel scoring system for response assessment at follow-up MRI in 10 patients with MIBC who underwent TURBT and neoadjuvant chemotherapy followed by radical cystectomy [21]. Hafeez et al. showed that quantitative DWI can be helpful for response assessment post radiotherapy in patients with MIBC [22]. We are not aware of any further studies which have investigated the diagnostic performance of MRI after cystoscopic intervention in a representative cohort with NMIBC and MIBC who underwent TURBT, cystectomy or long-term cystoscopic surveillance. Our results suggest that DWI can better distinguish residual tumour from acute inflammatory changes which are commonly encountered after TURBT, where the presence of mural oedema may cause high signal intensity on both T2 and DWI (‘T2-shine through’ effect), but can be mitigated by assessing the ADC map. In the longer term chronic inflammatory changes and fibrosis can also hinder assessment, but also typically do not show marked restricted diffusion. DWI is considered the dominant sequence for estimation of muscle invasion in VI-RADS [13]. Indeed, our findings are consistent with previous studies in the intervention naïve setting where DWI outperforms T2WI for tumour detection [23, 24]. Inter-reader agreement for tumour detection improved from fair (κ = 0.54) to moderate (κ = 0.70) when DWI was included. This was comparable to inter-reader agreement for VI-RADS shown by Barchetti et al. in the intervention-naïve bladder (κ = 0.73), which employed readers of similar experience [25].

Our study has some limitations including its retrospective nature. The sample size is relatively small due to the strict inclusion criteria employed, which was necessary to ensure our cohort benefited from more definitive follow-up histopathological assessment, however, this is comparable to a prior study [26]. The reference standard was TURBT or cystectomy. Despite this mixed reference standard, it was important to include patients with a representative spectrum of disease stages in the study, as this better reflects clinical practice where treatment choice depends on tumour stage and depth of invasion. Finally, we did not utilise dynamic contrast enhanced (DCE) imaging in this study. Although DCE is an integral component of multiparametric bladder MRI with VI-RADS, our study cohort pre-dated publication of VI-RADS, with our protocol at the time based on earlier work which suggested that addition of one functional sequence such as DWI was sufficient [23]. Furthermore VI-RADS was designed for treatment-naïve patients. A study by Wang et al. found that DWI was superior to DCE for differentiating recurrent bladder tumour from inflammation or fibrosis after TURBT [27]. Future studies in a larger population are warranted to investigate factors that affect accuracy of MRI after cystoscopic intervention with the goal of developing reliable and validated criteria for interpretation of MRI in this setting.

## Conclusion

Non-contrast MRI with DWI showed high sensitivity and relatively high specificity for detection of residual tumour after TURBT. Inter-reader agreement improved from fair to moderate with the addition of DWI. Our results highlight the usefulness of MRI in the post-TURBT setting to accurately detect residual disease and inform further management pathways.

### Supplementary Information

Below is the link to the electronic supplementary material.

Table 1 supplementary data: Diagnostic performance of MRI for tumour detection for each pathological T-category with T1WI and T2WI together and T1WI, T2WI and DWI combined for reader 1. Supplementary file1 (TIFF 70 kb)

## References

[1] Sung H, Ferlay J, Siegel RL et al (2021) Global cancer statistics 2020: GLOBOCAN estimates of incidence and mortality worldwide for 36 cancers in 185 countries. CA Cancer J Clin 71:209–249. 10.3322/caac.21660 33538338 10.3322/caac.21660

[2] Gandhi N, Krishna S, Booth CM et al (2018) Diagnostic accuracy of magnetic resonance imaging for tumour staging of bladder cancer: systematic review and meta-analysis. BJU Int 122(5):744–53. 10.1111/bju.14366 29727910 10.1111/bju.14366

[3] Babjuk M, Burger M, Capoun O et al (2022) European Association of Urology Guidelines on Non-muscle-invasive Bladder Cancer (Ta, T1, and Carcinoma in Situ). Eur Urol Jan;81(1):75–94. 10.1016/j.eururo.2021.08.010 34511303 10.1016/j.eururo.2021.08.010

[4] Flaig TW, Spiess PE, Abern M et al (2022) NCCN Guidelines insights: bladder cancer, version 2.2022. J Natl Compr Cancer Netw, 20 pp. 866-878 10.6004/jnccn.2022.0041 35948037

[5] Witjes JA, Bruins HM, Carrión A et al (2023) European Association of Urology Guidelines on Muscle-invasive and Metastatic Bladder Cancer: Summary of the 2023 Guidelines, Eur Urol Jan;85(1):17–31. 10.1016/j.eururo.2023.08.016 37858453 10.1016/j.eururo.2023.08.016

[6] Department of Health. NHS Cancer Reform Strategy. 2007.

[7] Independent Cancer Taskforce. Achieving world-class cancer outcomes - a strategy for England 2015–2020. CRUK Website; 2015. Available from: https://www.cancerresearchuk.org/sites/default/files/achieving_world-class_cancer_outcomes_-_a_strategy_for_england_2015-2020.pdf

[8] National Institute for Clinical Excellence surveillance of bladder cancer: diagnosis and management (NICE guideline NG2). 2019 https://www.nice.org.uk/guidance/ng2/resources/2019-surveillance-of-bladder-cancer-diagnosis-and-management-nice-guideline-ng2-pdf-8716335273925 31869030

[9] Shariat SF, Palpattu GS, Karakiewicz PI et al (2007) Discrepancy between clinical and pathologic staging: impact on prognosis after radical cystectomy. Eur Urol 51(1):137–151. 10.1016/j.eururo.2006.05.021 16793197 10.1016/j.eururo.2006.05.021

[10] Dutta SC, Smith Jr JA, Shappell SB et al (2001) Clinical under staging of high risk nonmuscle invasive urothelial carcinoma treated with radical cystectomy. J Urol Aug;166(2):490–3. 11458053 10.1016/S0022-5347(05)65969-1

[11] Harrison S, Briggs T, O’Flynn K. Urology: GIRFT Programme National Specialty Report, 1st ed. NHS England and NHS Improvement. 2018 https://gettingitrightfirsttime.co.uk/wp-content/uploads/2018/07/Urology-June18-M.pdf

[12] Bryan RT, Liu W, Pirrie SJ et al (2021) Comparing an imaging-guided pathway with the standard pathway for staging muscle-invasive bladder cancer: preliminary data from the BladderPath study. Eur Urol 80:12–5. 10.1016/j.eururo.2021.02.021 33653635 10.1016/j.eururo.2021.02.021

[13] Panebianco V, Narumi Y, Altun E et al (2018) Multiparametric Magnetic Resonance Imaging for Bladder Cancer: Development of VI-RADS (Vesical Imaging-Reporting And Data System). Eur Urol Sep;74(3):294–306. 10.1016/j.eururo.2018.04.029 29755006 10.1016/j.eururo.2018.04.029 PMC6690492

[14] Wang H, Luo C, Zhang F et al (2019) Multiparametric MRI for bladder cancer: Validation of VI-RADS for the detection of detrusor muscle invasion. Radiology 291: 668–674. https://doi.org/10.1148/radiol.2019182506 31012814 10.1148/radiol.2019182506

[15] El-Assmy A, Abou-El-Ghar ME, Refaie HF et al (2012) Diffusion weighted magnetic resonance imaging in follow-up of superficial urinary bladder carcinoma after transurethral resection: initial experience. BJU Int 110(11 Pt B):E622–7. 10.1111/j.1464-410X.2012.11345.x 10.1111/j.1464-410X.2012.11345.x 22757606

[16] van der Pol CB, Shinagare AB, Tirumani SH et al (2018) Bladder cancer local staging: multiparametric MRI performance following transurethral resection. Abdom Radiol 43:2412–2423. 10.1007/s00261-017-1449-0 10.1007/s00261-017-1449-0 29313114

[17] Lim CS, Tirumani S, van der Pol CB et al (2019) Use of quantitative T2-weighted and apparent diffusion coefficient texture features of bladder cancer and extravesical fat for local tumor staging after transurethral resection. AJR Am J Roentgenol 212:1060–1069. 10.2214/AJR.18.20718 30860885 10.2214/AJR.18.20718

[18] Amin M, Edge S, Greene F, et al AJCC Cancer Staging Manual, 8th edn. 2017 New York: Springer.

[19] Compérat E, Oszwald A, Wasinger G et al (2022) Updated pathology reporting standards for bladder cancer: biopsies, transurethral resections and radical cystectomies. World J Urol 40:915–927. 10.1007/s00345-021-03831-1 34554298 10.1007/s00345-021-03831-1 PMC8994708

[20] Sauter G, Algaba F, Amin M et al Tumours of the urinary system: non-invasive urothelial neoplasias. In: WHO classification of tumours of the urinary system and male genital organs. 2004 IARCC Press: Lyon.

[21] Pecoraro M, Del Giudice F, Magliocca F et al (2022) Vesical Imaging-Reporting and Data System (VI-RADS) for assessment of response to systemic therapy for bladder cancer: preliminary report. Abdom Radiol (NY) Feb;47(2):763–770. 10.1007/s00261-021-03365-5 34919160 10.1007/s00261-021-03365-5

[22] Hafeez S, Koh M, Jones K et al (2022) Assessing bladder radiotherapy response with quantitative diffusion-weighted magnetic resonance imaging analysis. Clin Oncol (R Coll Radiol) Oct;34(10):630–641. 10.1016/j.clon.2022.04.001 35534398 10.1016/j.clon.2022.04.001

[23] Takeuchi M, Sasaki S, Ito M et al (2009) Urinary bladder cancer: diffusion-weighted MR imaging--accuracy for diagnosing T stage and estimating histologic grade. Radiology Apr;251(1):112–121. 10.1148/radiol.2511080873 19332849 10.1148/radiol.2511080873

[24] Kobayashi S, Koga F, Yoshida S et al (2011) Diagnostic performance of diffusion-weighted magnetic resonance imaging in bladder cancer: potential utility of apparent diffusion coefficient values as a biomarker to predict clinical aggressiveness. Eur Radiol 21:2178–2186. 10.1007/s00330-011-2174-7 21688007 10.1007/s00330-011-2174-7

[25] Barchetti G, Simone G, Ceravolo I et al (2019) Multiparametric MRI of the bladder: inter-observer agreement and accuracy with the Vesical Imaging-Reporting and Data System (VI-RADS) at a single reference center. Eur Radiol Oct;29(10):5498–5506. 10.1007/s00330-019-06117-8 30887202 10.1007/s00330-019-06117-8

[26] Huele EH, Veenboer PW, Wessels FJ et al (2023) Value of multiparametric magnetic resonance imaging for local staging of invasive urinary bladder tumours. Urol Oncol Jan;41(1):49.e7-49.e12. 10.1016/j.urolonc.2022.09.026 36441069 10.1016/j.urolonc.2022.09.026

[27] Wang HJ, Pui MH, Guo Y et al (2014) Diffusion-weighted MRI in bladder carcinoma: the differentiation between tumor recurrence and benign changes after resection. Abdom Imaging 39(1):135–141. 10.1007/s00261-013-0038-0 24072383 10.1007/s00261-013-0038-0

