# Supplementary figures and images for: Evaluation of magnetic resonance imaging for bladder cancer detection following transurethral resection of bladder tumour (TURBT)
Source: Abdom Radiol (NY). 2024 May 8;49(7):2340–8. doi: 10.1007/s00261-024-04235-6 (PMC11286648; doi:10.1007/s00261-024-04235-6)

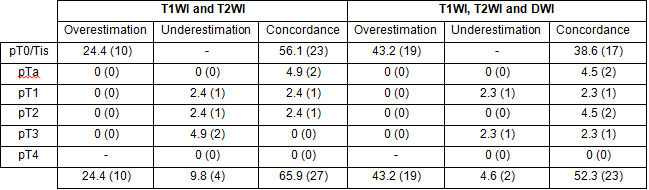

Supplement: Supplementary file 1 — Table 1 supplementary data: Diagnostic performance of MRI for tumour detection for each pathological T-category with T1WI and T2WI together and T1WI, T2WI and DWI combined for reader 1. Supplementary file1 (TIFF 70 kb) [file 261_2024_4235_MOESM1_ESM.tiff]
